# Supplementary material for: Altered tRNA dynamics during translocation on slippery mRNA as determinant of spontaneous ribosome frameshifting
Source: Nat Commun. 2022 Jul 22;13:4231. doi: 10.1038/s41467-022-31852-w (PMC9307594; doi:10.1038/s41467-022-31852-w)
Supplement: Supplementary file 1 — Supplementary Information [file 41467_2022_31852_MOESM1_ESM.pdf]

Supplementary Figures

Altered tRNA dynamics during translocation on slippery mRNA as determinant of spontaneous ribosome frameshifting

Panagiotis Poulis[1], Anoshi Patel[1,2], Marina V. Rodnina[1] & Sarah Adio[2]*

[1]Department of Physical Biochemistry, Max Planck Institute for Multidisciplinary Sciences, Göttingen, Germany

[2]Department of Molecular Structural Biology, Institute for Microbiology and Genetics, Georg-August University of Göttingen, Göttingen, Germany

*correspondence to sarah.adio@uni-goettingen.de

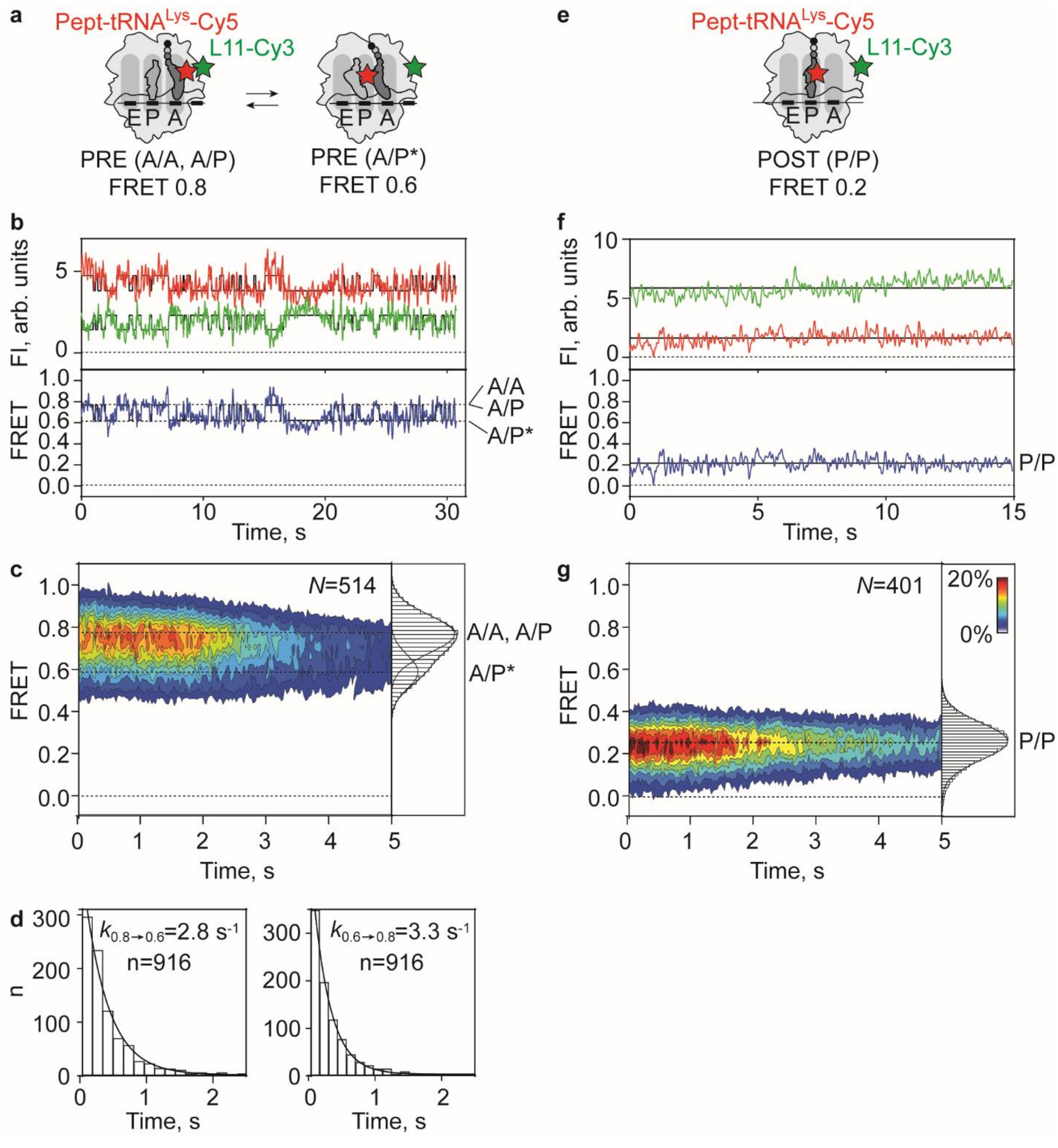

Supplementary Figure 1. L11-tRNA smFRET in PRE and POST complexes on slippery mRNA.

(a) Schematic of the smFRET experiment monitoring the conformation of pept-tRNA^{Lys}-Cy5 (Cy5; red star) relative to L11-Cy3 (green star) on PRE complexes. Assignment of smFRET efficiencies to A/A and A/P (FRET 0.8) and the A/P* (FRET 0.6) is based on previous smFRET experiments[1, 2, 3] and recent cryo-EM structures[4, 5, 6]. In the structures, the distance between U47-Cy5 in the elbow of tRNA^{Lys} and L11(C38-Cy3) is similar in A/A and A/P conformations (~50 Å), but increases in the A/P* (~60 Å).

(b) Representative time traces showing pept-tRNA fluctuations between the A/A, A/P and A/P* conformations in PRE complexes. Here, and in all other smFRET traces the time courses of Cy3- and Cy5-

fluorescence intensity (FI; arb. units, arbitrary units) and FRET are shown in green, red and blue, respectively.

(c) Contour plot and histogram showing the distribution of FRET values in PRE complexes. Data are from 3 independent experiments (N=3). Transition frequency, i.e. the number of transitions from A/A or A/P to A/P* and A/P* to A/A or A/P, is 5.5±0.7 per trace.

(d) Transition rates and fits of fluctuations between FRET 0.8 (A/A, A/P) and 0.6 (A/P*) (left panel) and between FRET 0.6 (A/P*) and 0.8 (A/A, A/P) (right panel) (Table 1). n, number of transitions.

(e) Schematic of the smFRET experiment monitoring the position of pept-tRNA^{Lys}-Cy5 (Cy5; red star) relative to L11-Cy3 (green star) on POST complexes.

(f) Representative time traces showing Cy3 (green) and Cy5 (red) fluorescence intensity and FRET (blue) corresponding to the P/P tRNA conformation in POST complexes.

(g) Contour plot and histogram showing the distribution of FRET values in POST complexes. Data are from 3 independent experiments (N=3).

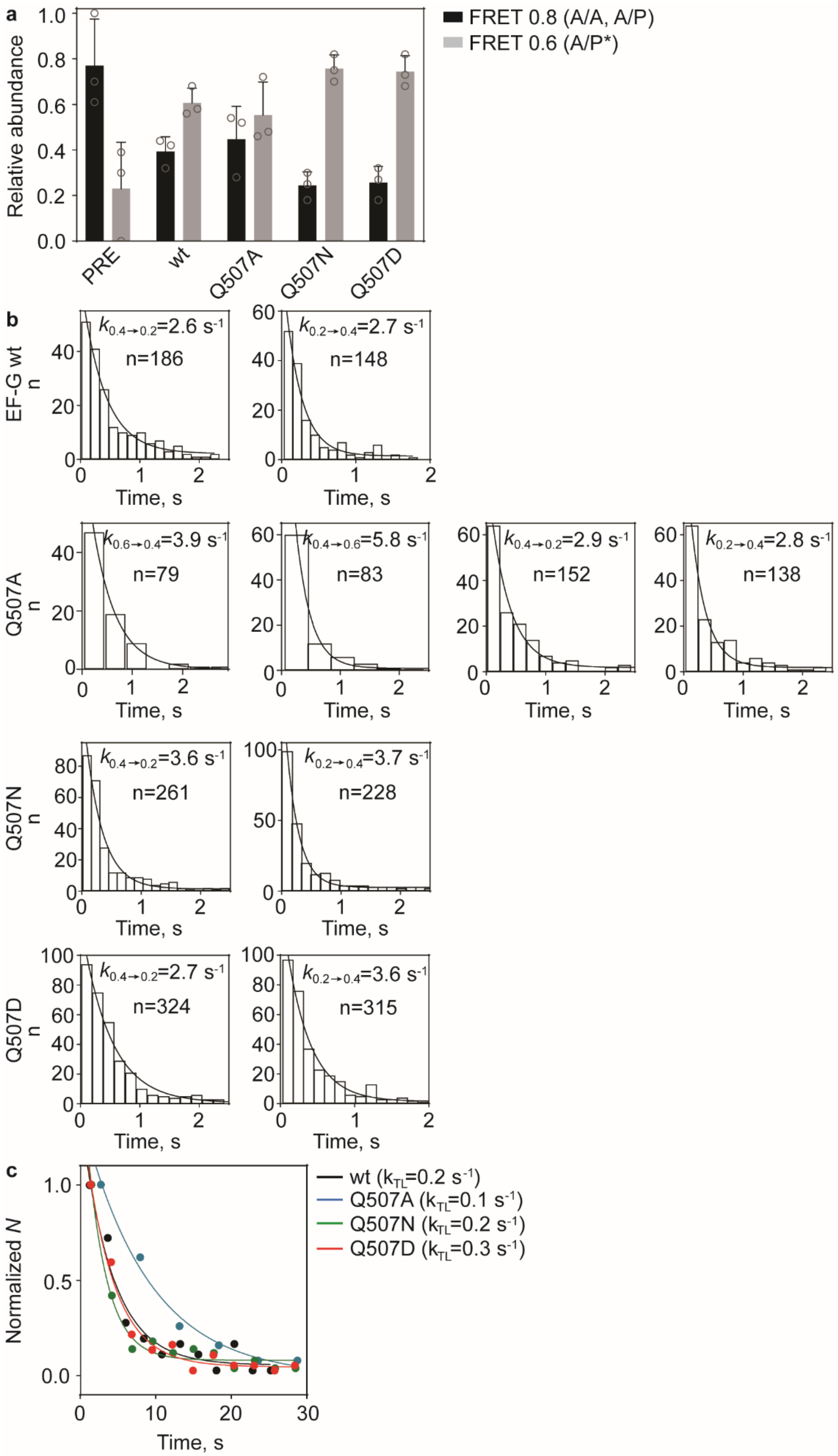
a
FRET 0.8 (A/A, A/P)
FRET 0.6 (A/P*)
Relative abundance
PRE
wt
Q507A
Q507N
Q507D
b
EF-G wt
Q507A
Q507N
Q507D
n
Time, s
k0.4→0.2=2.6 s-1
n=186
k0.2→0.4=2.7 s-1
n=148
k0.6→0.4=3.9 s-1
n=79
k0.4→0.6=5.8 s-1
n=83
k0.4→0.2=2.9 s-1
n=152
k0.2→0.4=2.8 s-1
n=138
k0.4→0.2=3.6 s-1
n=261
k0.2→0.4=3.7 s-1
n=228
k0.4→0.2=2.7 s-1
n=324
k0.2→0.4=3.6 s-1
n=315
c
Normalized N
Time, s
wt (kTL=0.2 s-1)
Q507A (kTL=0.1 s-1)
Q507N (kTL=0.2 s-1)
Q507D (kTL=0.3 s-1)

Supplementary Figure 2. Pept-tRNA translocation on slippery mRNA.

(a) Distribution of A/A, A/P (black) and A/P* (grey) states within 33 ms before translocation with EF-G(wt), EF-G(Q507A), EF-G(Q507N) and EF-G(Q507D), compared to the PRE complex in the absence of EF-G. Data are presented as mean ± s.d. from at least 3 independent experiments (N=3 for PRE, N=12 for EF-G(wt), N=4 for Q507A, N=5 for Q507N and N=9 for Q507D).

(b) Transition rates and single exponential fits of fluctuations between FRET 0.6 (A/P*), 0.4 (CHI), and 0.2 (P/P) states during translocation on slippery mRNA with EF-G(wt), EF-G(Q507A), EF-G(Q507N), and EF-G(Q507D) (Table 1). n, number of transitions.

(c) Translocation rates and curve fits on slippery mRNA mediated by EF-G(wt) (black), EF-G(Q507A) (blue), EF-G(Q507N) (green) and EF-G(Q507D) (red) on the fraction of ribosomes that show CHI state fluctuations (Table 1). Normalization was performed by division by the maximum value of the histogram.

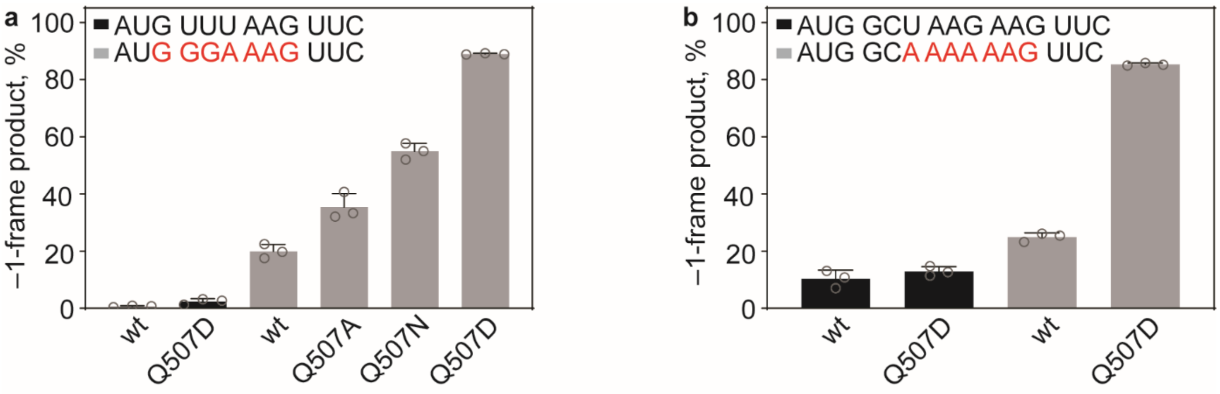

Supplementary Figure 3. Frameshifting during translation on the slippery and non-slippery mRNA.

(a), (b) Frameshifting on non-slippery (black columns) and slippery (grey columns) mRNA in the presence of EF-G(wt) or EF-G Q507 mutants. The 0- and –1-frame products were quantified by HPLC analysis of translation products[7]. Shown are mean values from 3 independent experiments (N=3) and error bars represent the standard deviation.

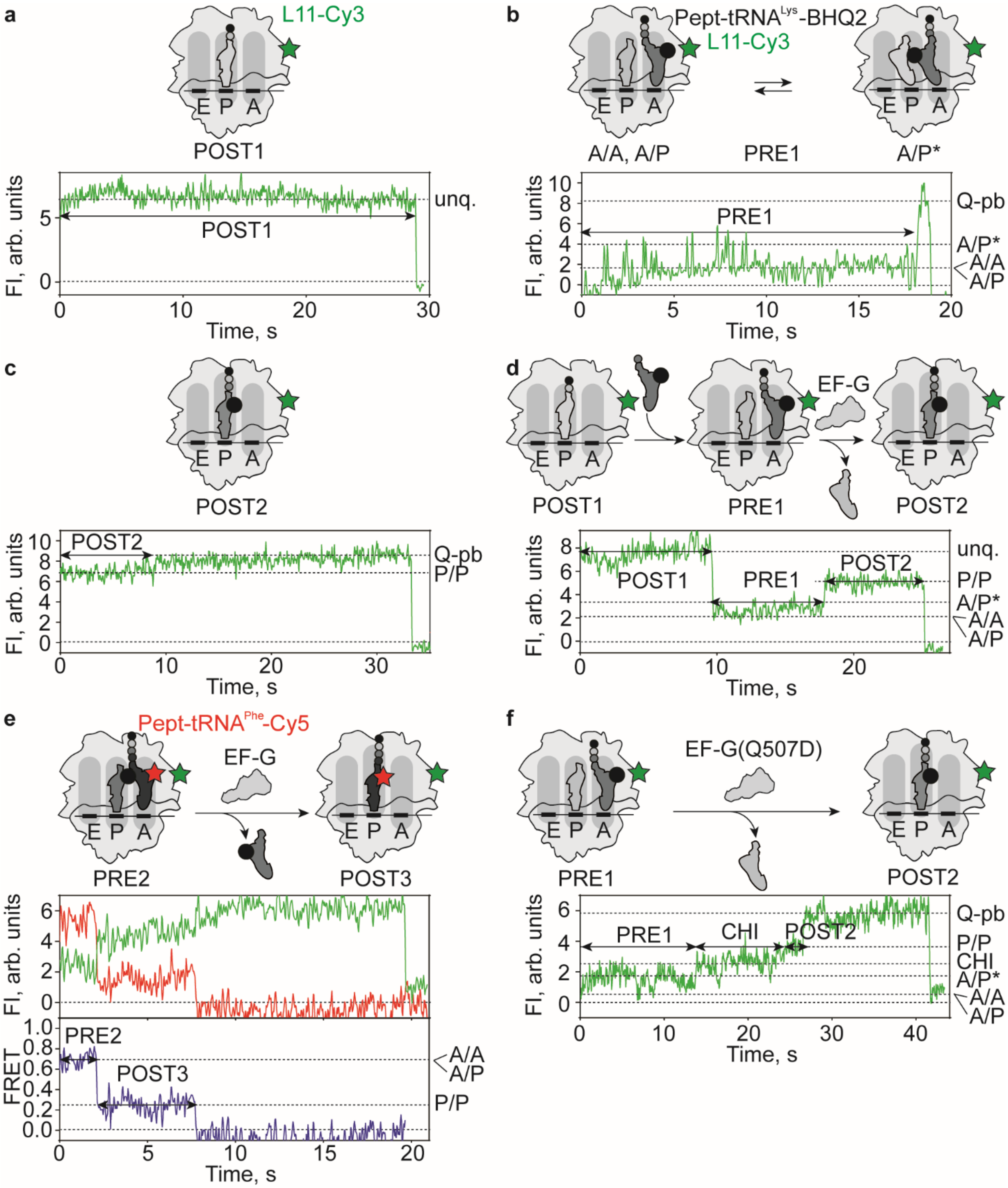

Supplementary Figure 4. Assignment of L11-Cy3 fluorescence intensity levels to pept-tRNA^{Lys}-BHQ2 states.

(a) Schematic of the L11-Cy3 POST1 complex with pept-tRNA^{Gly} in the P site and a representative time trace of L11-Cy3 fluorescence (FI) in the absence of tRNA^{Phe}-Cy5 (*N*=270). unq, unquenched FI of L11-Cy3.

(b) Schematic of the L11-Cy3 PRE1 complex with pept-tRNA^{Lys}-BHQ2 (black circle) in the A site and deacylated tRNA^{Gly} in the P site and time trace of L11-Cy3 showing fluorescence fluctuations between A/A, A/P or A/P* (*N*=130). Q-pb, BHQ2 photobleaching.

(c) Schematic of the L11-Cy3 POST2 complex with pept-tRNA^{Lys}-BHQ2 in the P site and time trace of L11-Cy3 in the P/P state (*N*=75). POST2 was formed by incubation of PRE2 complexes with EF-G(wt) prior to immobilization.

(d) Schematic and time trace of pept-tRNA^{Lys}-BHQ2 binding to POST1 complexes and rapid (in one frame) translocation from the A to the P site by EF-G(wt) on non-slippery mRNA (*N*=89). Lys-tRNA^{Lys}-BHQ2 was added in complex with EF-Tu and GTP together with EF-G(wt) to immobilized POST1 complexes.

(e) Schematic of translocation of PRE2 forming the POST3 complex and time trace showing FI of L11-Cy3 (green) and pept-tRNA^{Phe}-Cy5 (red) and FRET (blue) during pept-tRNA^{Phe}-Cy5 movement from the A to the P site (within one frame) by EF-G(wt) on non-slippery mRNA. PRE2 was formed by incubation of immobilized PRE1 with EF-G(wt) and Phe-tRNA^{Phe}-Cy5 prior to imaging.

(f) Schematic and time trace of slow pept-tRNA^{Lys}-BHQ2 translocation from the A to the P site by EF-G(Q507D) on slippery mRNA (*N*=37).

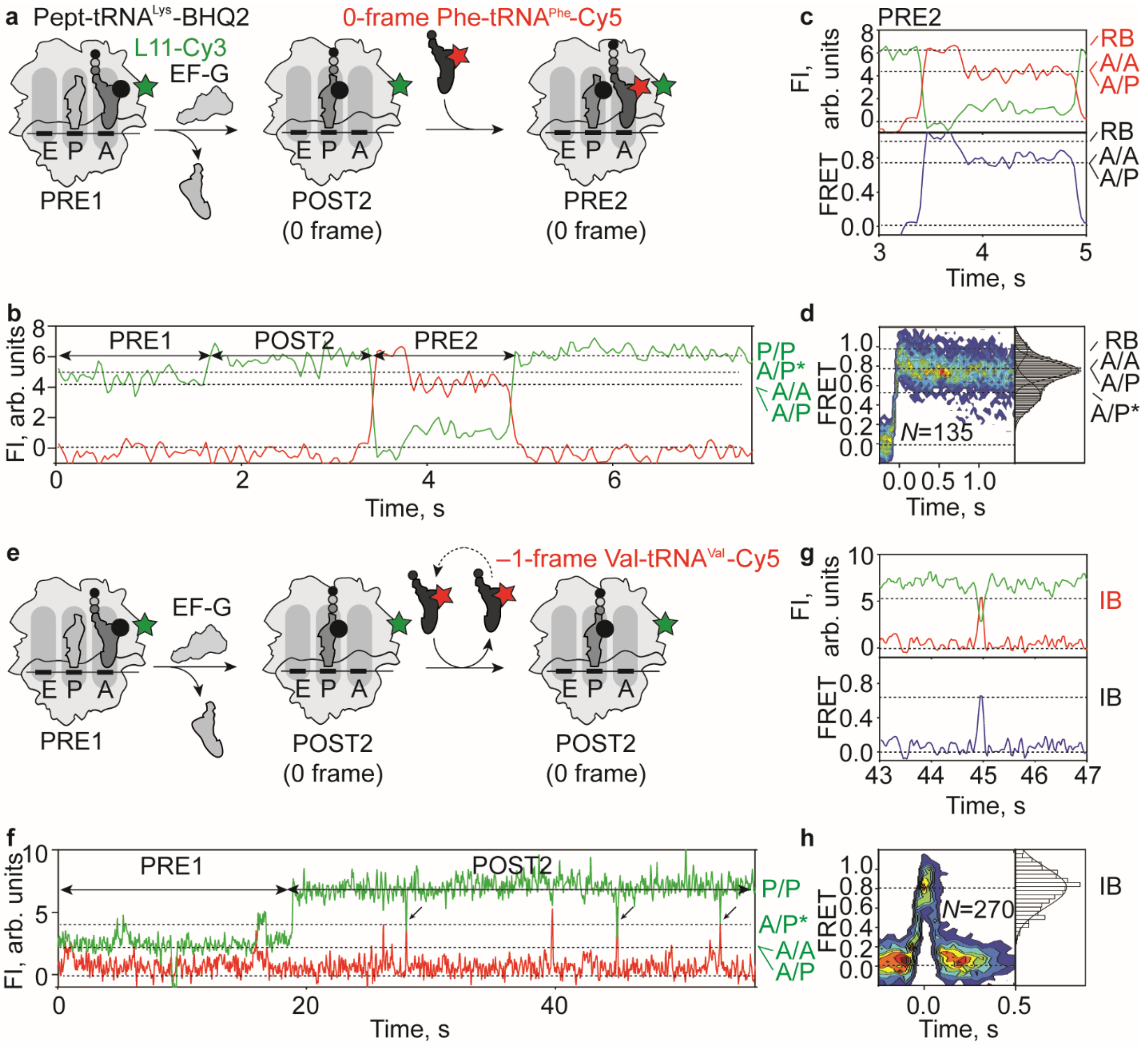

Supplementary Figure 5: Translocation of pept-tRNA^{Lys} on non-slippery mRNA, incorporation of 0- and rejection of –1-frame aa-tRNAs.

(a) Schematic of translocation of pept-tRNA^{Lys}-BHQ2 (black circle) from the A to the P site on non-slippery mRNA by EF-G(wt) and subsequent accommodation of the cognate 0-frame Phe-tRNA^{Phe}-Cy5 (red star). EF-G(wt) was added to immobilized PRE1 complexes together with the EF-Tu–GTP–Phe-tRNA^{Phe}-Cy5 complex.

(b) Representative time trace of pept-tRNA^{Lys}-BHQ2 movement and accommodation of Phe-tRNA^{Phe}-Cy5 (box) (*N*=42). FI, fluorescence intensity. Green labels at the right Y-axis indicate tRNA^{Lys} conformational states monitored by Cy3 fluorescence, which are assigned in Supplementary Fig. 4.

(c) Zoom-in into Supplementary Fig. 5b showing Cy3 and Cy5 FI and calculated FRET of Phe-tRNA^{Phe}-Cy5 binding to POST2 forming PRE2 complexes. The FRET changes are consistent with previously characterized steps of ribosome binding (RB) and tRNA accommodation in the A site[3]. Phe-tRNA^{Phe}-Cy5 accommodation

results in rapid peptide bond formation followed by fluctuations of pept-tRNA^{Phe}-Cy5 between A/A, A/P and A/P* states (see Supplementary Fig. 1). Red labels indicate conformational states of Phe-tRNA^{Phe}-Cy5.

(d) Contour plot showing distribution of FRET values after Phe-tRNA^{Phe}-Cy5 incorporation into the POST2 complex forming PRE2. Traces were synchronized to the point with FRET>0. Histogram at the right shows FRET distribution after post-synchronization.

(e) Schematic of translocation of pept-tRNA^{Lys}-BHQ2 from the A to the P site on non-slippery mRNA by EF-G(wt) and sampling of POST2 complexes by the non-cognate –1-frame Val-tRNA^{Val}-Cy5 (red star). EF-G(wt) was added to immobilized PRE1 complexes together with the EF-Tu-Val-tRNA^{Val}-Cy5-GTP complex.

(f) Representative time trace of pept-tRNA^{Lys}-BHQ2 translocation (one frame) and subsequent sampling of POST2 complexes by the near-cognate –1-frame tRNA^{Val}-Cy5 (arrows) (N=68).

(g) Zoom-in into Supplementary Fig. 5f showing Cy3 and Cy5 FI and calculated FRET of initial binding (IB) without accommodation of –1-frame Val-tRNA^{Val}-Cy5 to POST2 complexes.

(h) Contour plot showing distribution of FRET values during Val-tRNA^{Val}-Cy5 sampling of POST2 complexes. Traces were synchronized to the point with FRET>0. Histogram at the right shows FRET distribution after post-synchronization.

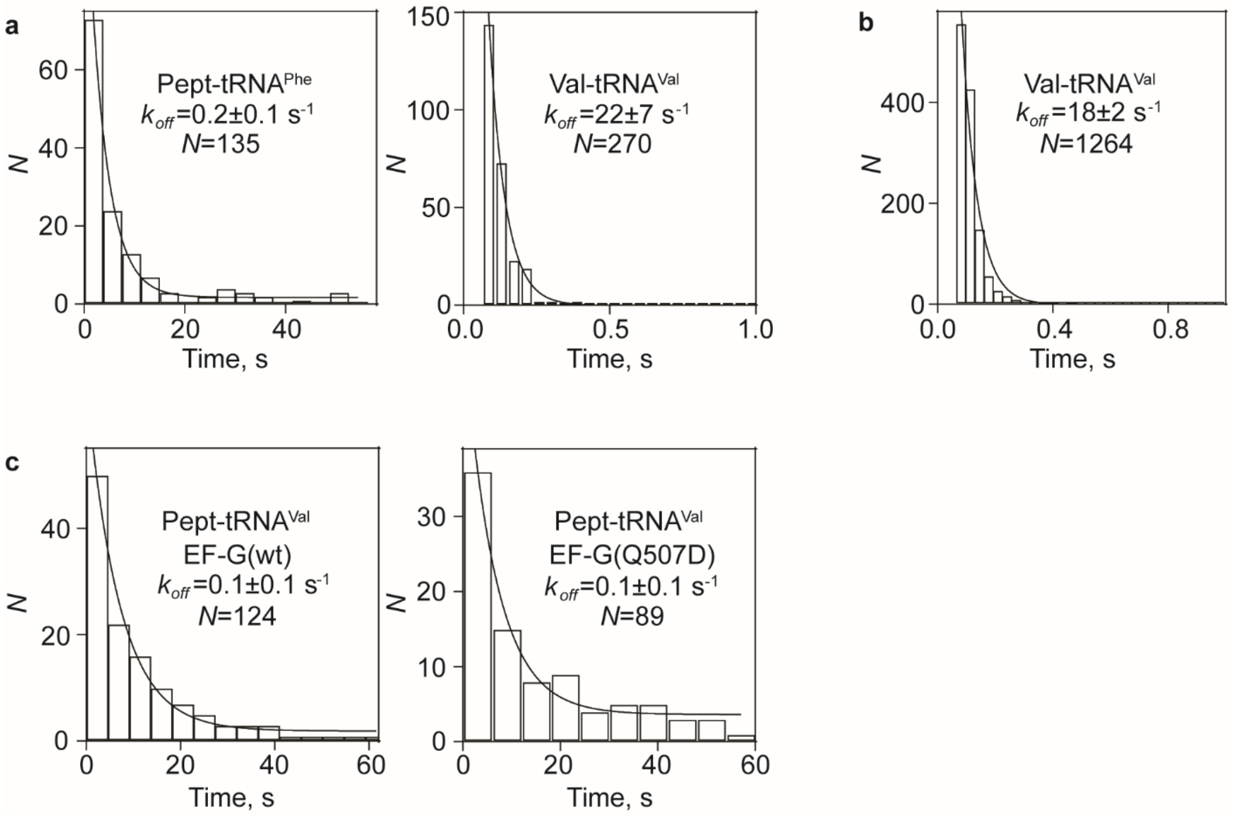

Supplementary Figure 6. Binding of 0- and –1-frame tRNAs to PRE2 or POST2 complexes with tRNA^{Lys}-BH2 in the P site formed on non-slippery and slippery mRNA

(a) Dwell-time distribution of pept-tRNA^{Phe}-Cy5 accommodated on PRE2 complexes (left panel) compared to transient binding of Val-tRNA^{Val}-Cy5 on POST2 complexes (right panel) formed on non-slippery mRNA with EF-G(wt) (from Supplementary Fig. 5d and h, respectively). k_{off} values and s.d. were obtained by single exponential fitting (black lines).

(b) Dwell-time distribution of Val-tRNA^{Val}-Cy5 on POST2 complex formed on slippery mRNA after rapid translocation with EF-G(wt) (from Fig. 3d).

(c) Dwell-time distribution of pept-tRNA^{Val}-Cy5 on PRE2 complexes formed on slippery mRNA after slow translocation with EF-G (wt) (left panel, from Fig. 3h) or EF-G(Q507D) (right pane, from Fig. 3l).

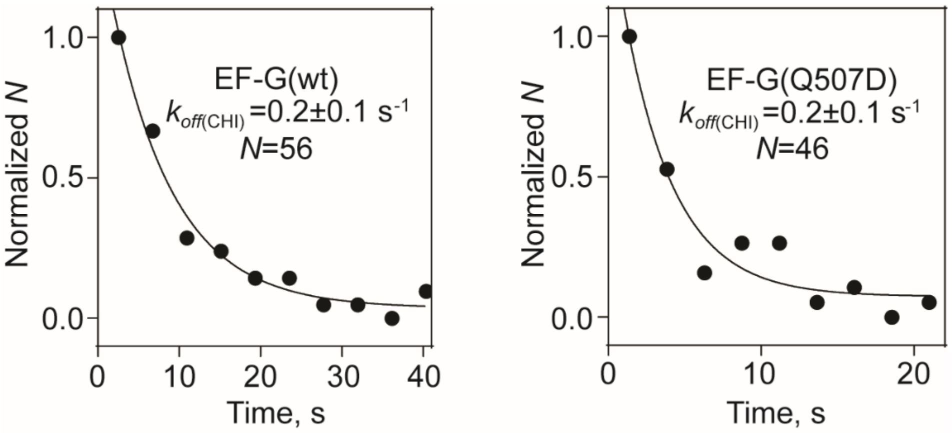

Supplementary Figure 7. Dwell times of the CHI state during slow translocation of pept-tRNA^{Lys}-BHQ2 on slippery mRNA.

Dwell time distribution of slow translocation intermediate on slippery mRNA by EF-G(wt) (Fig. 3f) or EF-G(Q507D) (Fig. 3j). k_{off} values and s.d. were obtained by single exponential fitting (black lines). Normalization was performed by division by the maximum value of the histogram.

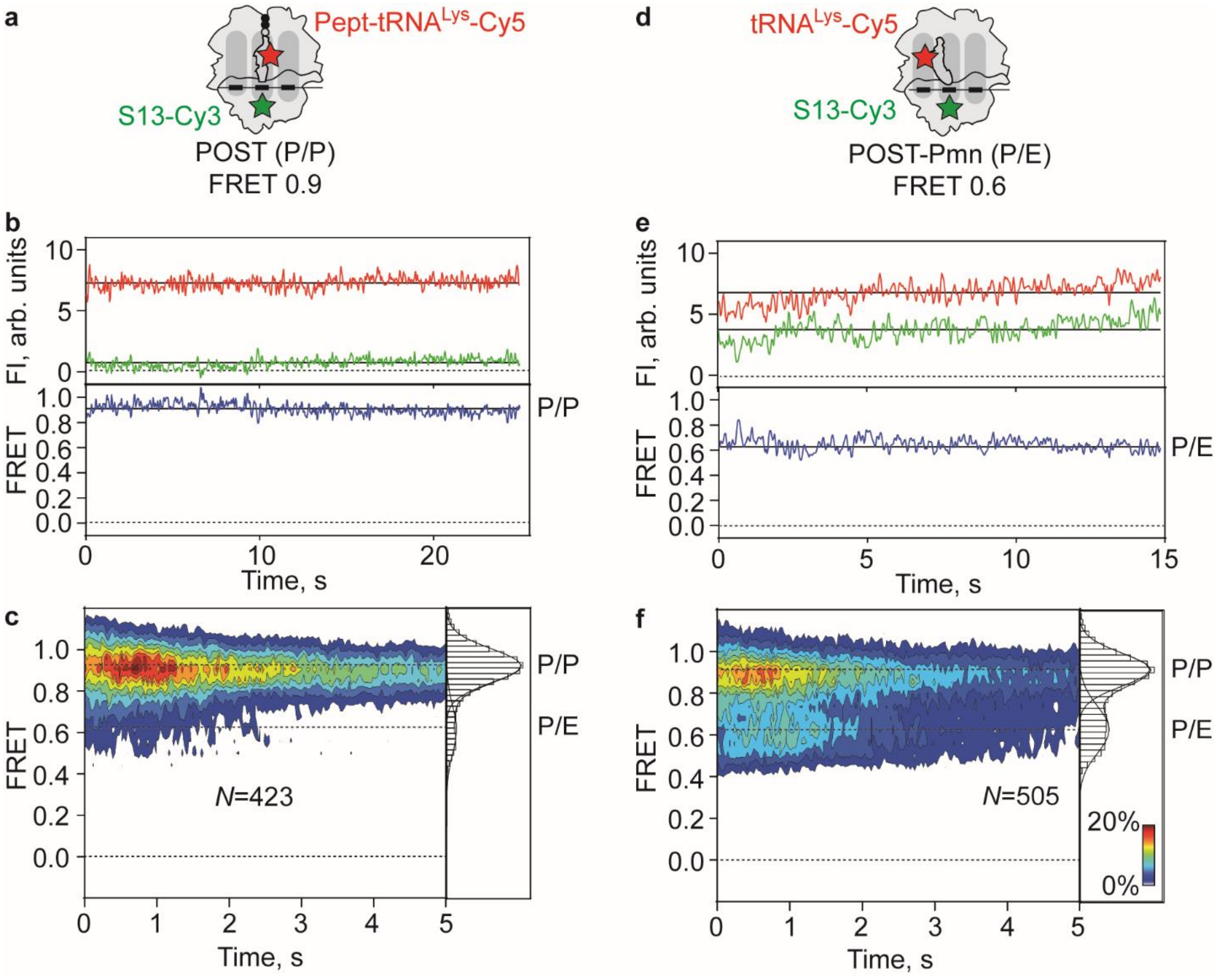

Supplementary Figure 8. Assignment of S13-tRNA smFRET efficiencies to tRNA states.

(a) Schematic of the experiment monitoring smFRET between pept-tRNA^{Lys}-Cy5 (Cy5; red star) and ribosomal protein S13-Cy3 (green star) in POST complexes.

(b) Representative time trace with FRET 0.9 corresponding to the P/P state of tRNA in the POST complex.

(c) Contour plot and histogram showing the distribution of FRET values in the POST complex. Data are from 4 independent experiments (N=4).

(d) Schematic of the smFRET experiment using deacylated tRNA^{Lys}-Cy5 (Cy5; red star) and the ribosomal protein S13-Cy3 (green star) in POST complexes. To obtain deacylated tRNA^{Lys} in the P site, POST fMAK-tRNA^{Lys} complex was treated with puromycin (Pmn, 1mM).

(e) Representative trace with FRET 0.6 corresponding to the P/E state of tRNA.

(f) Contour plot and histogram showing two distinct populations with FRET 0.9 and 0.6 corresponding to P/P and P/E states in POST-Pmn complexes. Data are from 4 independent experiments (N=4). Transition frequency, i.e. the number of transitions from P/P to P/E and P/E to P/P is 0.4±0.1 per trace.

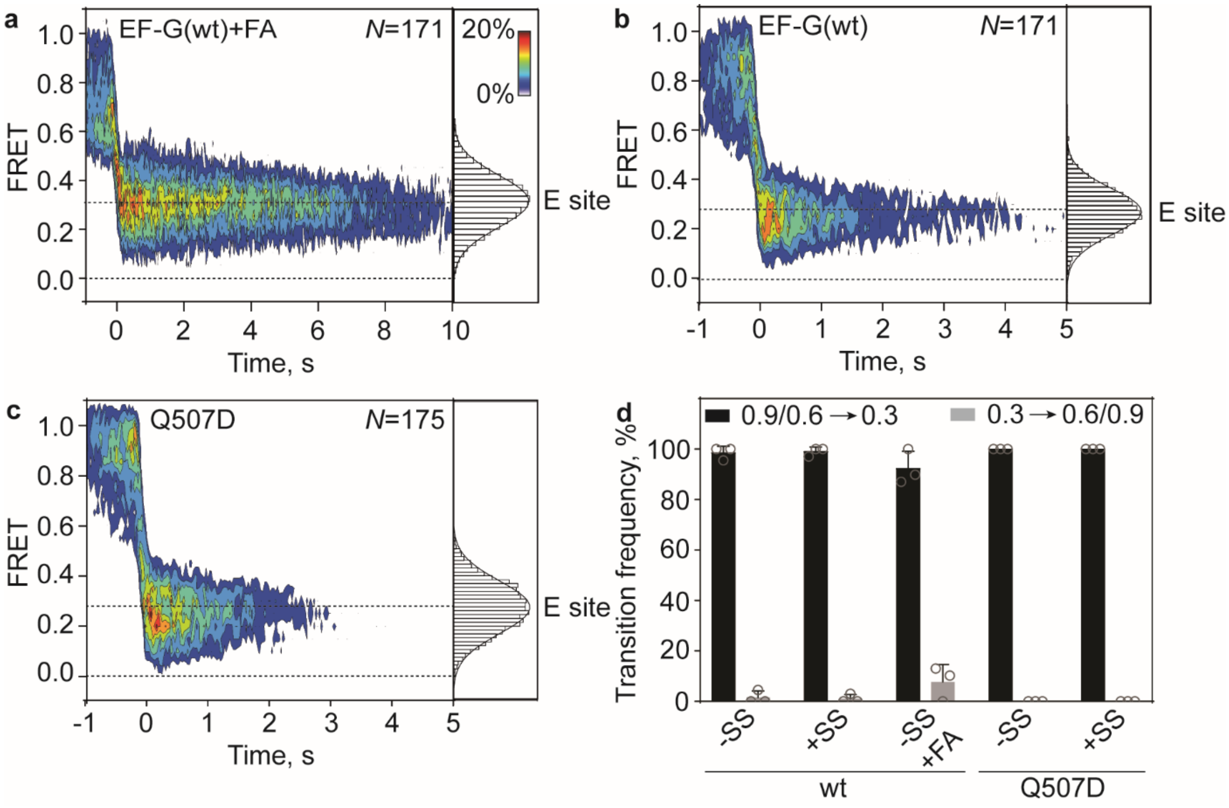

Supplementary Figure 9. Displacement of deacylated tRNA upon translocation.

(a) Assignment of the FRET 0.3 state. Contour plot shows the distribution of FRET values during translocation on non-slippery mRNA mediated by EF-G(wt) in the presence of fusidic acid (FA, 200 µM), which delays the dissociation of deacylated tRNA from the ribosome due to retention of EF-G[8, 9]. Traces are found in FRET 0.9 (P/P) or FRET 0.6 (P/E) (Supplementary Fig. 4) before entering a long-lived state with FRET 0.3, which we assign as the E site. Histogram at the right shows distribution of FRET values after synchronization to the first transition below FRET 0.5. The duration of the FRET 0.3 state is limited by photobleaching (Methods). Data are from 3 independent experiments (N=3).

(b), (c) Contour plots showing the distribution of FRET values during translocation on slippery mRNA mediated by EF-G(wt) and EF-G(Q507D). Note that the transition from FRET 0.3 to FRET 0.0 is much faster than in (a), suggesting that it is due to tRNA dissociation from the ribosome rather than photobleaching. Histogram at the right show distribution of FRET values after synchronization to the first transition below FRET 0.5. Data are from 3 independent experiments (N=3).

(d) Transition frequency between FRET states during translocation on non-slippery and slippery mRNA with EF-G(wt) and EF-G(Q507D). After addition of EF-G, we observe transitions exclusively from FRET 0.9 (P/P) or 0.6 (P/E) to FRET 0.3 (E) state (black), and no reverse transition to PRE states (grey). Data are presented as mean ± s.d. from at least 3 independent experiments (N=4 for EF-G(wt)/-SS, N=3 for EF-G(wt)/+SS, N=3 for EF-G(wt)/-SS/+FA, N=3 for EF-G(Q507D)/-SS), N=3 for EF-G(Q507D)/+SS).

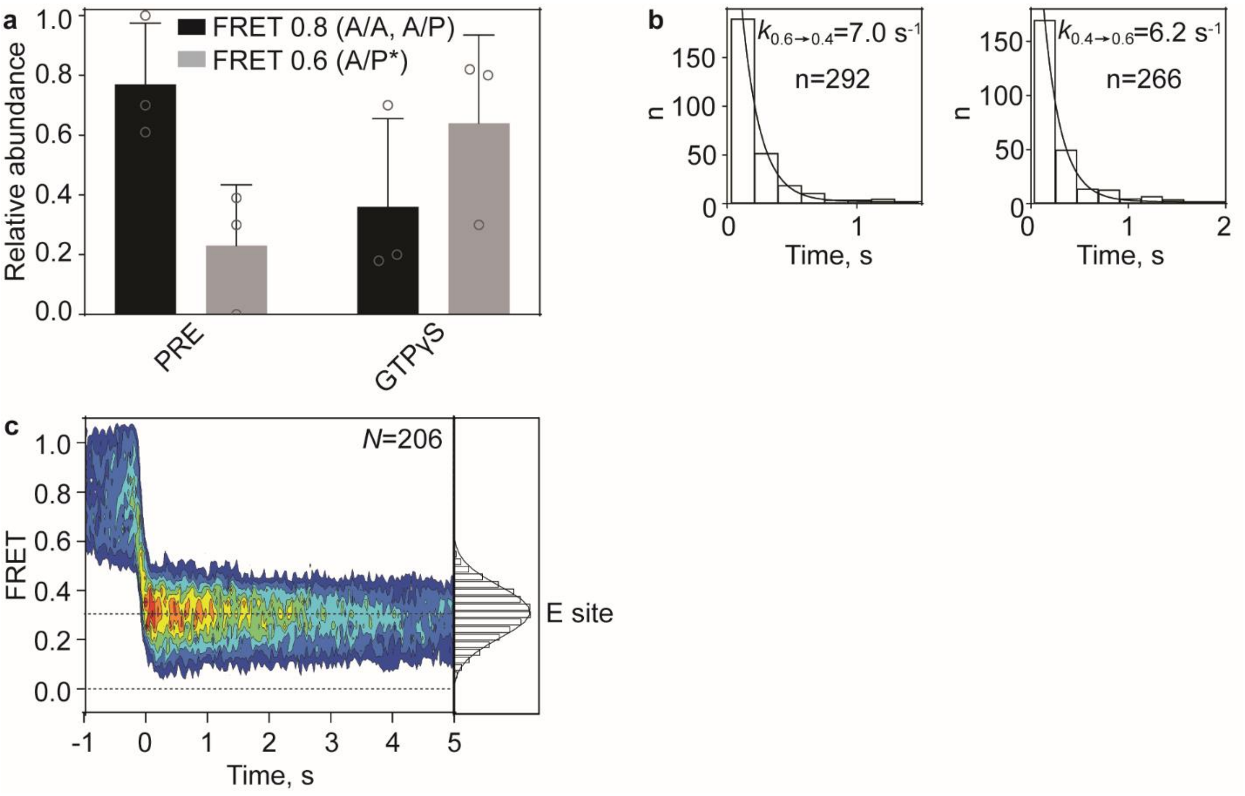

Supplementary Figure 10. Analysis of translocation in the presence of EF-G(wt)—GTPγS.

(a) Distribution of A/A, A/P (black) and A/P* (grey) states within 33 ms before translocation of pept-tRNA on slippery mRNA by EF-G(wt)—GTPγS, compared to the PRE complex in the absence of EF-G. Data are presented as mean ± s.d. from 6 independent experiments (N=6).

(b) Transition rates and fits of fluctuations between FRET 0.6 (A/P*) to 0.4 (CHI) state (left panel) and of the FRET 0.4 (CHI) to the 0.6 (A/P*) state (right panel) during translocation of pept-tRNA on slippery mRNA mediated by EF-G(wt)—GTPγS (Table 1).

(c) Contour plot showing the distribution of S13-tRNA FRET values during translocation on non-slippery mRNA mediated by EF-G(wt)—GTPγS. Traces fluctuate between FRET 0.9 (P/P) and FRET 0.6 (P/E) before entering a long-lived state with FRET 0.3 (E site). Histogram at the right shows distribution of FRET values after synchronization to the first transition below FRET 0.5. Data are from 3 independent experiments (N=3).

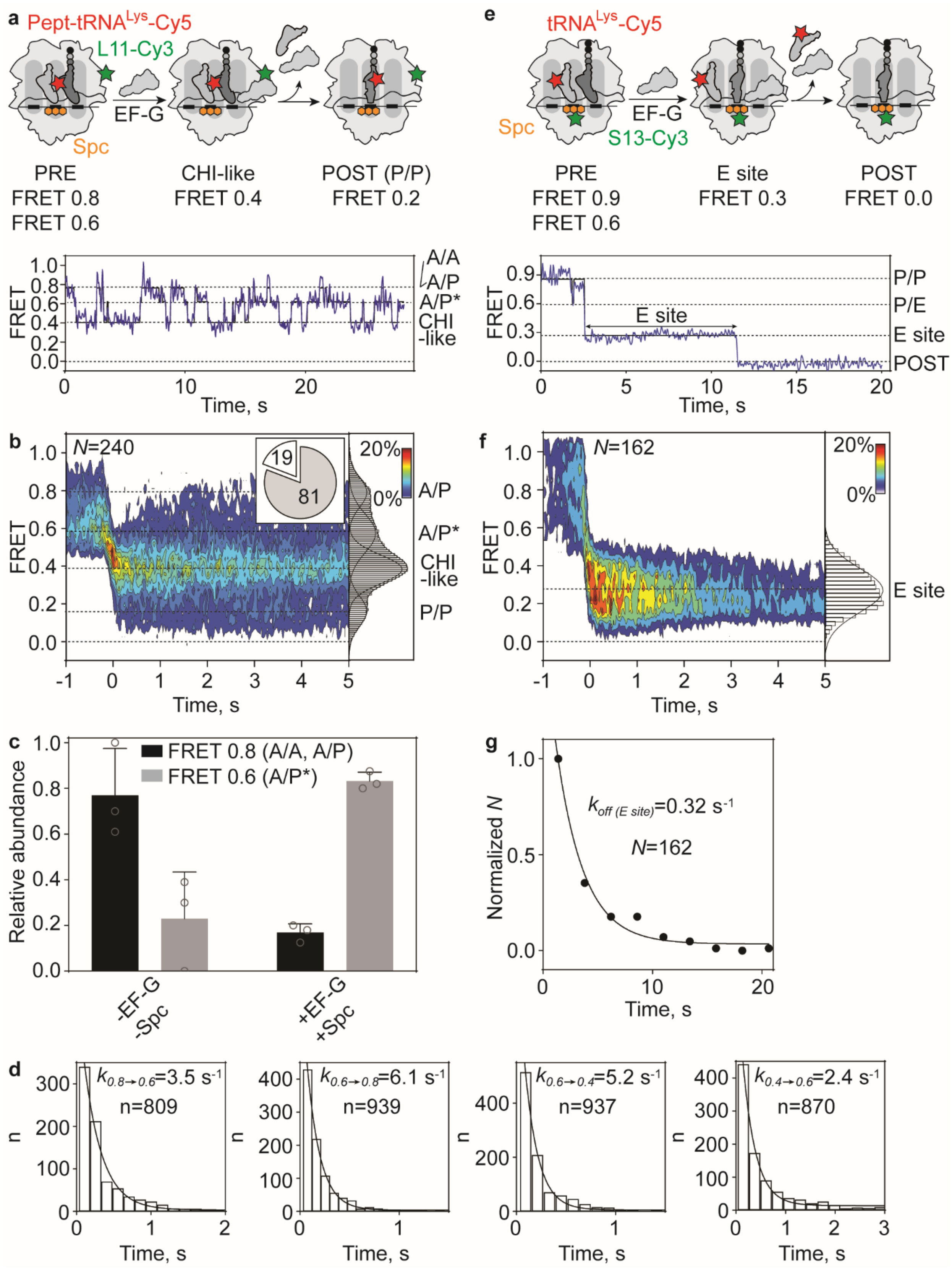

Supplementary Figure 11. Translocation on slippery mRNA in the presence of Spc.

(a) Above: schematic of smFRET experiment monitoring movement of pept-$tRNA^{Lys}$-Cy5 (red stars) relative to L11-Cy3 (green star) in the presence of Spc (orange). Below: representative smFRET time trace of pept-tRNA translocation on slippery mRNA with EF-G(wt) in the presence of Spc. Trace shows fluctuations between FRET 0.8 (A/A, A/P), 0.6 (A/P*) and 0.4 (CHI-like).

(b) Contour plot showing distribution of FRET values during translocation of pept-tRNA on slippery mRNA by EF-G(wt) in the presence of Spc. 19% of traces show direct transitions from A/A, A/P or A/P* to P/P, while 81% samples CHI-like state. Traces are synchronized to the first transition with FRET≤0.5. Histogram at the right shows distribution of FRET values after synchronization. Data are from 3 independent experiments (N=3).

(c) Distribution of A/A, A/P (black) and A/P* (grey) states within 33 ms before translocation of pept-tRNA on slippery mRNA by EF-G(wt) in the presence of Spc, compared to PRE complex. Data are presented as mean ± s.d. from 3 independent experiments (N=3).

(d) Transition rates and curve fits of fluctuations from FRET 0.8 (A/A, A/P) to the 0.6 (A/P*) and from FRET 0.6 to 0.4 (CHI) state during translocation of pept-tRNA on slippery mRNA mediated EF-G(wt) in the presence of Spc (Table 1).

(e) Above: schematic of smFRET experiment monitoring the movement of $tRNA^{Lys}$-Cy5 (red star) relative to protein S13-Cy3 (green star) during translocation by EF-G(wt) in the presence of Spc. Below: representative smFRET time trace for tRNA translocation. Trace shows stepwise transition from FRET 0.9 (P/P) or 0.6 (P/E) to 0.3 (E) to 0.0 (dissociation).

(f) Contour plot showing distribution of FRET values during translocation of deacylated tRNA on slippery mRNA by EF-G(wt) in the presence of Spc. Traces were synchronized to the first transition below FRET 0.5. Histogram at the right shows distribution of FRET values after synchronization.

(g) Rate and curve fit of tRNA dissociation from the E site on slippery mRNA in the presence of Spc (Table 2). Normalization was performed by division by the maximum value of the histogram. Data are from 3 independent experiments (N=3).

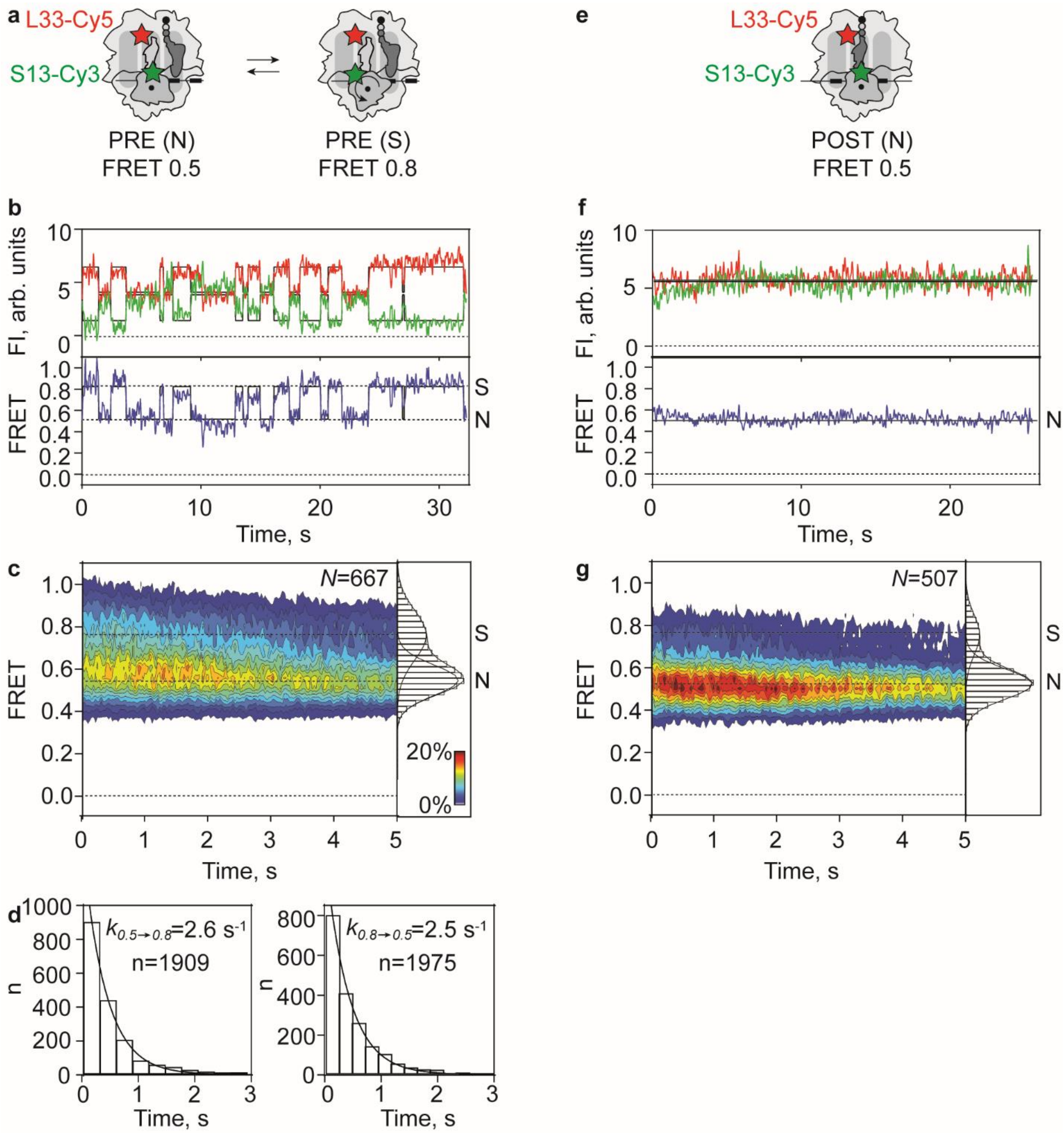

Supplementary Figure 12. S13-L33 smFRET in PRE and POST complexes formed on slippery mRNA.

(a) Schematic of the smFRET experiment monitoring the movement of the SSU head domain in PRE complexes formed on mRNA with the slippery sequence G GGA AAG using reporters on S13-Cy3 (green star) and L33-Cy5 (red star).

(b) Representative smFRET time trace corresponding to SSU head domain movements between the swiveled (S) and non-swiveled (N) states of the PRE complex with FRET 0.8 and FRET 0.5, respectively.

(c) Contour plot and histogram showing the distribution of FRET values in the PRE complex. Data are from 3 independent experiments (N=3). Transition frequency, i.e. the number of transitions from S to N and N to S is 6.3±0.4 per trace.

(d) Transition rates between FRET 0.5 (N) and 0.8 (S) (left panel) and between FRET 0.8 (S) and 0.5 (N) (right panel) in the absence of EF-G (Table 3). n, number of transitions.

(e) Schematic of the smFRET experiment monitoring the movement of the SSU head domain in POST complexes using reporters on S13-Cy3 (green star) and L33-Cy5 (red star).

(f) Representative trace corresponding to the non-swiveled (N) conformation of the SSU head with FRET 0.5.

(g) Contour plot and histogram showing the distribution of FRET values in the POST complex. Data are from three independent experiments (N=3).

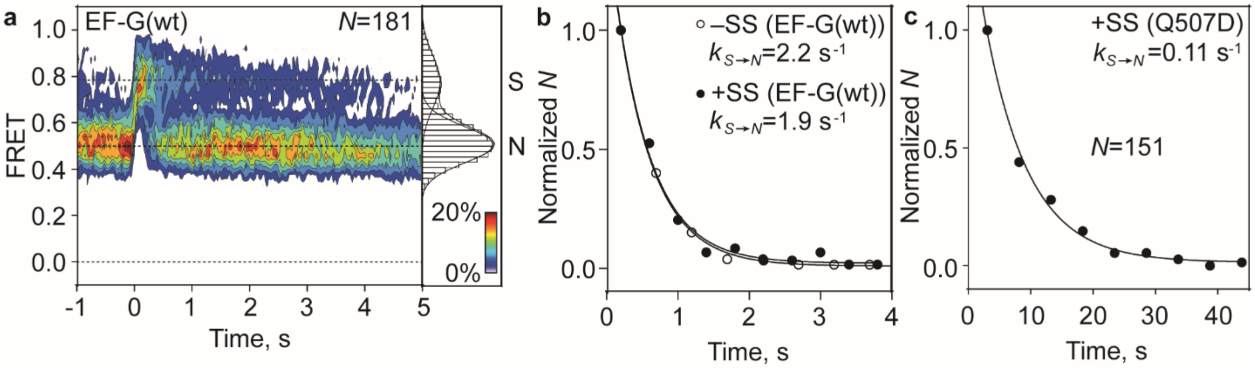

Supplementary Figure 13. SSU head swiveling during translocation by EF-G(wt) and EF-G(Q507D).

(a) Contour plot showing the distribution of FRET values during translocation on non-slippery mRNA mediated by EF-G(wt). Traces were synchronized at the last transition to FRET 0.8 (S) state. Histogram at the right shows distribution of FRET values after synchronization. Data are from 5 independent experiments (N=5).

(b) Transition rate and curve fit of the last FRET 0.8 (S) state before transition to stable FRET 0.5 (N) during translocation on non-slippery (open circles) and slippery (closed circles) mRNA by EF-G(wt) (Table 3). Normalization was performed by division by the maximum value of the histogram.

(c) Transition rate and curve fit of the last FRET 0.8 (S) state during translocation on slippery mRNA by EF-G(Q507D) (Table 3). Normalization was performed by division by the maximum value of the histogram.

Supplementary References

1. Adio S, Senyushkina T, Peske F, Fischer N, Wintermeyer W, Rodnina MV. Fluctuations between multiple EF-G-induced chimeric tRNA states during translocation on the ribosome. *Nat Commun* **6**, 7442 (2015).

2. Chen C*, et al.* Single-molecule fluorescence measurements of ribosomal translocation dynamics. *Mol Cell* **42**, 367-377 (2011).

3. Geggier P*, et al.* Conformational sampling of aminoacyl-tRNA during selection on the bacterial ribosome. *J Mol Biol* **399**, 576-595 (2010).

4. Rundlet EJ*, et al.* Structural basis of early translocation events on the ribosome. *Nature* **595**, 741-745 (2021).

5. Carbone CE, Loveland AB, Gamper HB, Jr., Hou YM, Demo G, Korostelev AA. Time-resolved cryo-EM visualizes ribosomal translocation with EF-G and GTP. *Nat Commun* **12**, 7236 (2021).

6. Petrychenko V, Peng BZ, de A. P. Schwarzer AC, Peske F, Rodnina MV, Fischer N. Structural mechanism of GTPase-powered ribosome-tRNA movement. *Nat Commun* **12**, 5933 (2021).

7. Peng BZ, Bock LV, Belardinelli R, Peske F, Grubmuller H, Rodnina MV. Active role of elongation factor G in maintaining the mRNA reading frame during translation. *Sci Adv* **5**, eaax8030 (2019).

8. Belardinelli R, Rodnina MV. Effect of Fusidic Acid on the Kinetics of Molecular Motions During EF-G-Induced Translocation on the Ribosome. *Sci Rep* **7**, 10536 (2017).

9. Wasserman MR, Alejo JL, Altman RB, Blanchard SC. Multiperspective smFRET reveals rate-determining late intermediates of ribosomal translocation. *Nat Struct Mol Biol* **23**, 333-341 (2016).
