# Supplementary material for: Altered tRNA dynamics during translocation on slippery mRNA as determinant of spontaneous ribosome frameshifting
Source: Nat Commun. 2022 Jul 22;13:4231. doi: 10.1038/s41467-022-31852-w (PMC9307594; doi:10.1038/s41467-022-31852-w)
Supplement: Supplementary file 3 — Reporting Summary [file 41467_2022_31852_MOESM3_ESM.pdf]

nature portfolio

Corresponding author(s): SA

Last updated by author(s): 20/06/2022

Reporting Summary

Nature Portfolio wishes to improve the reproducibility of the work that we publish. This form provides structure for consistency and transparency in reporting. For further information on Nature Portfolio policies, see our Editorial Policies and the Editorial Policy Checklist.

Statistics

For all statistical analyses, confirm that the following items are present in the figure legend, table legend, main text, or Methods section.

n/a	Confirmed	
☐	☒	The exact sample size (*n*) for each experimental group/condition, given as a discrete number and unit of measurement
☐	☒	A statement on whether measurements were taken from distinct samples or whether the same sample was measured repeatedly
☒	☐	The statistical test(s) used AND whether they are one- or two-sided<br>*Only common tests should be described solely by name; describe more complex techniques in the Methods section.*
☒	☐	A description of all covariates tested
☒	☐	A description of any assumptions or corrections, such as tests of normality and adjustment for multiple comparisons
☐	☒	A full description of the statistical parameters including central tendency (e.g. means) or other basic estimates (e.g. regression coefficient) AND variation (e.g. standard deviation) or associated estimates of uncertainty (e.g. confidence intervals)
☒	☐	For null hypothesis testing, the test statistic (e.g. *F*, *t*, *r*) with confidence intervals, effect sizes, degrees of freedom and *P* value noted<br>*Give P values as exact values whenever suitable.*
☒	☐	For Bayesian analysis, information on the choice of priors and Markov chain Monte Carlo settings
☒	☐	For hierarchical and complex designs, identification of the appropriate level for tests and full reporting of outcomes
☒	☐	Estimates of effect sizes (e.g. Cohen's *d*, Pearson's *r*), indicating how they were calculated

Our web collection on statistics for biologists contains articles on many of the points above.

Software and code

Policy information about availability of computer code

Data collection	Single-molecule FRET data was collected using xCellence rt imaging software from Olympus Corporation.
Data analysis	Single-molecule data analysis was performed using a custom script written in the Matlab (version 2011b, Mathworks) software, based on the built-in Statistics Toolbox and is described in Adio et al., Nat Commun, 2015. The Matlab scripts used in this study are available upon request. Kinetic analysis of FRET time traces by Hidden Markov fitting was performed using open-source vbFRET software (http://vbfret.sourceforge.net/). Commercially available Graphpad Prism 8 was used for analysis and representation of frameshifting data.

For manuscripts utilizing custom algorithms or software that are central to the research but not yet described in published literature, software must be made available to editors and reviewers. We strongly encourage code deposition in a community repository (e.g. GitHub). See the Nature Portfolio guidelines for submitting code & software for further information.

Data

Policy information about availability of data

All manuscripts must include a data availability statement. This statement should provide the following information, where applicable:

- Accession codes, unique identifiers, or web links for publicly available datasets
- A description of any restrictions on data availability
- For clinical datasets or third party data, please ensure that the statement adheres to our policy

All data are shown in the main and supplementary figures. Due to the lack of a public repository for smFRET data, data are available from the corresponding author upon reasonable request.

Human research participants

Policy information about studies involving human research participants and Sex and Gender in Research.

Reporting on sex and gender	n/a
Population characteristics	n/a
Recruitment	n/a
Ethics oversight	n/a

Note that full information on the approval of the study protocol must also be provided in the manuscript.

Field-specific reporting

Please select the one below that is the best fit for your research. If you are not sure, read the appropriate sections before making your selection.

☒ Life sciences ☐ Behavioural & social sciences ☐ Ecological, evolutionary & environmental sciences

For a reference copy of the document with all sections, see nature.com/documents/nr-reporting-summary-flat.pdf

Life sciences study design

All studies must disclose on these points even when the disclosure is negative.

Sample size	The sample size for single-molecule FRET experiments was determined by analysis of 3000 to 10000 fluorescent spots showing Cy3 and Cy5 fluorescence obtained in at least three independent experiments. The sample size is sufficient because the R2 values of Gaussian and exponential fits of the data are better than 0.96 throughout the study.
Data exclusions	Fluorescence artifacts were excluded using criteria outlined in the methods. Exclusion criteria was pre-established as the correlation Cy3 and Cy5 signals.
Replication	All findings were successfully replicated at least three times using either reagents from a single preparation or from the standard preparation protocol.
Randomization	Experiments were not randomized and not relevant to this study. All experiments were performed with biochemically purified reagents that passed the internal quality control. Randomization is not relevant because all the ribosomes used in this study were subjected to the same experimental treatment. NO humans were involved in this study.
Blinding	Not applicable. Blinding is not relevant in this study because NO human subjects were involved.

Reporting for specific materials, systems and methods

We require information from authors about some types of materials, experimental systems and methods used in many studies. Here, indicate whether each material, system or method listed is relevant to your study. If you are not sure if a list item applies to your research, read the appropriate section before selecting a response.

Materials & experimental systems

n/a	Involved in the study
☐	☐ Antibodies
☐	☐ Eukaryotic cell lines
☐	☐ Palaeontology and archaeology
☐	☐ Animals and other organisms
☐	☐ Clinical data
☐	☐ Dual use research of concern

Methods

n/a	Involved in the study
☐	☐ ChIP-seq
☐	☐ Flow cytometry
☐	☐ MRI-based neuroimaging

Antibodies

Antibodies used: n/a

Validation: n/a

Eukaryotic cell lines

Policy information about cell lines and Sex and Gender in Research

Cell line source(s): n/a

Authentication: n/a

Mycoplasma contamination: n/a

Commonly misidentified lines (See ICLAC register): n/a

Palaeontology and Archaeology

Specimen provenance: n/a

Specimen deposition: n/a

Dating methods: n/a

☐ Tick this box to confirm that the raw and calibrated dates are available in the paper or in Supplementary Information.

Ethics oversight: n/a

Note that full information on the approval of the study protocol must also be provided in the manuscript.

Animals and other research organisms

Policy information about studies involving animals; ARRIVE guidelines recommended for reporting animal research, and Sex and Gender in Research

Laboratory animals: n/a

Wild animals: n/a

Reporting on sex: n/a

Field-collected samples: n/a

Ethics oversight: n/a

Note that full information on the approval of the study protocol must also be provided in the manuscript.

Clinical data

Policy information about clinical studies

All manuscripts should comply with the ICMJE guidelines for publication of clinical research and a completed CONSORT checklist must be included with all submissions.

Clinical trial registration	n/a
Study protocol	n/a
Data collection	n/a
Outcomes	n/a

Dual use research of concern

Policy information about dual use research of concern

Hazards

Could the accidental, deliberate or reckless misuse of agents or technologies generated in the work, or the application of information presented in the manuscript, pose a threat to:

No	Yes	
☒	☐	Public health
☒	☐	National security
☒	☐	Crops and/or livestock
☒	☐	Ecosystems
☒	☐	Any other significant area

Experiments of concern

Does the work involve any of these experiments of concern:

No	Yes	
☒	☐	Demonstrate how to render a vaccine ineffective
☒	☐	Confer resistance to therapeutically useful antibiotics or antiviral agents
☒	☐	Enhance the virulence of a pathogen or render a nonpathogen virulent
☒	☐	Increase transmissibility of a pathogen
☒	☐	Alter the host range of a pathogen
☒	☐	Enable evasion of diagnostic/detection modalities
☒	☐	Enable the weaponization of a biological agent or toxin
☒	☐	Any other potentially harmful combination of experiments and agents

ChIP-seq

Data deposition

☐ Confirm that both raw and final processed data have been deposited in a public database such as GEO.

☐ Confirm that you have deposited or provided access to graph files (e.g. BED files) for the called peaks.

Data access links<br>*May remain private before publication.*	n/a
Files in database submission	n/a
Genome browser session<br>(e.g. UCSC)	n/a

Methodology

Replicates	n/a
Sequencing depth	n/a

Antibodies	n/a
Peak calling parameters	n/a
Data quality	n/a
Software	n/a

Flow Cytometry

Plots

Confirm that:

- [ ] The axis labels state the marker and fluorochrome used (e.g. CD4-FITC).
- [ ] The axis scales are clearly visible. Include numbers along axes only for bottom left plot of group (a 'group' is an analysis of identical markers).
- [ ] All plots are contour plots with outliers or pseudocolor plots.
- [ ] A numerical value for number of cells or percentage (with statistics) is provided.

Methodology

Sample preparation	n/a
Instrument	n/a
Software	n/a
Cell population abundance	n/a
Gating strategy	n/a

- [ ] Tick this box to confirm that a figure exemplifying the gating strategy is provided in the Supplementary Information.

Magnetic resonance imaging

Experimental design

Design type	n/a
Design specifications	n/a
Behavioral performance measures	n/a

Acquisition

Imaging type(s)	n/a
Field strength	n/a
Sequence & imaging parameters	n/a
Area of acquisition	n/a

Diffusion MRI ☐ Used ☐ Not used

Preprocessing

Preprocessing software	n/a
Normalization	n/a
Normalization template	n/a
Noise and artifact removal	n/a
Volume censoring	n/a

Statistical modeling & inference

Model type and settings: n/a

Effect(s) tested: n/a

Specify type of analysis: ☐ Whole brain ☐ ROI-based ☐ Both

Statistic type for inference (See Eklund et al. 2016): n/a

Correction: n/a

Models & analysis

n/a	Involved in the study
☐	☐ Functional and/or effective connectivity
☐	☐ Graph analysis
☐	☐ Multivariate modeling or predictive analysis

Functional and/or effective connectivity: n/a

Graph analysis: n/a

Multivariate modeling and predictive analysis: n/a
